# Spontaneous tricuspid valve chordal rupture in a dog with severe, irreversible pulmonary hypertension caused by *Angiostrongylus vasorum* infection

**DOI:** 10.1186/s12917-020-02531-z

**Published:** 2020-08-26

**Authors:** Viktor Szatmári

**Affiliations:** Department of Clinical Sciences, Faculty of Veterinary Medicine, Utrecht University, Yalelaan 108, 3584 CM Utrecht, The Netherlands

**Keywords:** Ascites, Congestive heart failure, Fenbendazole, Flail leaflet, Heartworm, Mitral stenosis, Sildenafil

## Abstract

**Background:**

The adult worms of *Angiostrongylus vasorum* reside in the pulmonary artery of dogs and can lead to cardiac, respiratory, and central neurologic signs. Due to luminal obstruction and perivascular inflammation of the pulmonary artery branches, pulmonary hypertension can arise. Pulmonary hypertension, in turn, can lead to severe damage of the right-sided cardiac structures, leading to right ventricular remodeling and tricuspid valve regurgitation.

**Case presentation:**

An 8-year-old neutered female English Cocker Spaniel was presented to the author’s institution because of abdominal distention and exercise intolerance. Ascites caused by congestive right-sided heart failure was found to be responsible for these problems. The underlying etiology of the right-sided heart failure was a severe pulmonary hypertension caused by *Angiostrongylus vasorum* infection. Echocardiography revealed, in addition to a severe concentric and eccentric right ventricular hypertrophy, right atrial and pulmonary trunk dilation, severe tricuspid valve regurgitation, and a systolic flail of the anterior leaflet of the tricuspid valve, resulting from ruptured chordae tendineae. As a coincidental finding, a congenital mitral stenosis was found. Oral therapy was initiated with daily administration of fenbendazole for 2 weeks along with daily administration of oral sildenafil until the re-check examination. At the 6-week re-check the dog showed full clinical and partial echocardiographic recovery, and both the blood antigen test for *Angiostrongylus vasorum* and the fecal Baermann larva isolation test were negative. When the sildenafil therapy was ceased after tapering the daily dosage, the owner reported recurrence of abdominal distension. Re-starting the sildenafil therapy resulted in resolution of this problem. The dog was reported to be clinically healthy with daily sildenafil administration 7 months after the initial presentation.

**Conclusions:**

The present case report describes a dog where angiostrongylosis led to congestive right-sided heart failure resulting from severe pulmonary hypertension. The secondary right ventricular eccentric hypertrophy together with suspected papillary muscular ischemia were the suspected cause of the ruptured major tricuspid chordae tendineae, which led to a severe tricuspid valve regurgitation. Despite eradication of the worms, the severe pulmonary hypertension persisted. Treatment with daily oral sildenafil, a pulmonary arterial vasodilator, was enough to keep the dog free of clinically apparent ascites.

## Background

*Angiostrongylus vasorum*, also known as the French heartworm, is a metastrongyloid nematode, whose adults live in the pulmonary artery of dogs, foxes and other canids [1]. Dogs infected with this worm can be either asymptomatic or show mild to life-threatening clinical signs [1, 2]. Most clinical signs of the infected dogs are the direct or indirect results of either coagulopathy or pulmonary pathology and can manifest in central neurologic, respiratory or cardiovascular signs or hemorrhagic diathesis [2]. Due to luminal obstruction and perivascular inflammatory changes of the pulmonary artery branches caused directly by the parasites, pre-capillary pulmonary hypertension can arise [3]. Pulmonary hypertension will subsequently lead to pressure overload of the right ventricle, which will result in right ventricular concentric and eccentric hypertrophy [3–5]. Via annulus dilatation the right ventricular eccentric hypertrophy can cause tricuspid valve regurgitation [3–5]. Clinical signs related to the cardiac and pulmonary pathology include exercise intolerance and syncope, both resulting from decreased cardiac output, and ascites resulting from congestive right-sided heart failure [3].

The tricuspid valve apparatus consists of the right atrioventricular annulus, the tricuspid valve leaflets, chordae tendineae and papillary muscles [6]. The tricuspid valve leaflets are adjoined to the papillary muscles or directly to the right ventricular wall by the chordae tendineae, which are fibrous chords [6]. The chordae tendineae together with the papillary muscles are parts of the so called “tensor apparatus” of the right atrioventricular valve, which is responsible for systolic closure of the valve, thereby preventing tricuspid valve prolapse and regurgitation [6]. The major component of the chords is collagen (80%) and the rest is elastin and endothelial cells [6]. Regurgitation of the tricuspid and pulmonic valves is considered to be physiologic in several mammalian species, including dogs, as long as it is trivial to mild on color Doppler echocardiographic images, and causes no right atrial or right ventricular enlargement, nor an audible murmur on cardiac auscultation [5, 7]. A more than physiologic regurgitation typically results in a right-sided apical systolic murmur [4].

The present case report describes a flail tricuspid valve leaflet as a result of ruptured chordae tendineae. Though rupture of chordae tendineae of the mitral valve is a common finding in dogs and humans as a result of myxomatous mitral valve degeneration, ruptured chordae tendineae of the tricuspid valve has not yet been reported in dogs, and it is an extremely rare finding in humans [8–11].

## Case presentation

An 8 years old, 17.1 kg, neutered female English Cocker Spaniel was presented to the cardiology service of the author’s institution for evaluation of abdominal distension. This problem, along with exercise intolerance, became apparent to the owners a week prior to presentation. In addition, a mild cough during excitement of about 2 weeks duration was reported. The referring veterinarian performed a diagnostic abdominocentesis and afterwards referred the dog to a mobile ultrasound service for an echocardiogram. Based on the results of these examinations congestive right-sided heart failure as a result of pulmonary hypertension was suspected to be the cause of the abdominal distension. Subsequently, the referring veterinarian submitted a blood sample to a veterinary laboratory for an antigen test for *Angiostrongylus vasorum.* This test was positive. Treatment with oral furosemide (20 mg/dog BID) and oral milbemycin (milbemycin oxime 12.5 mg with praziquantel 125 mg/dog once a week) was started. Because of the lack of clinical improvement during the first several days of therapy, the dog was referred to the cardiology service of the author’s clinic. The dog had never been to endemic areas of *Dirofilaria immitis* and it was not known to have had any history of trauma.

At presentation the dog was bright, alert and responsive with a body condition score of 6 out of 9. Abdominal distension was apparent with a positive fluid wave. No signs of labored breathing were noticed. The respiratory rate was 24 breaths/min and the breathing pattern was costo-abdominal. The femoral pulse was moderately powerful, regular, symmetric, and without a pulse deficit, with a frequency of 108/min. Mucous membranes were pink with a capillary refill time within 1 s. A grade 4 out of 6 systolic cardiac murmur was auscultated over the right cardiac apex. No jugular venous distension was observed; however, the hepato-jugular reflux test was positive.

**Fig. 1:**
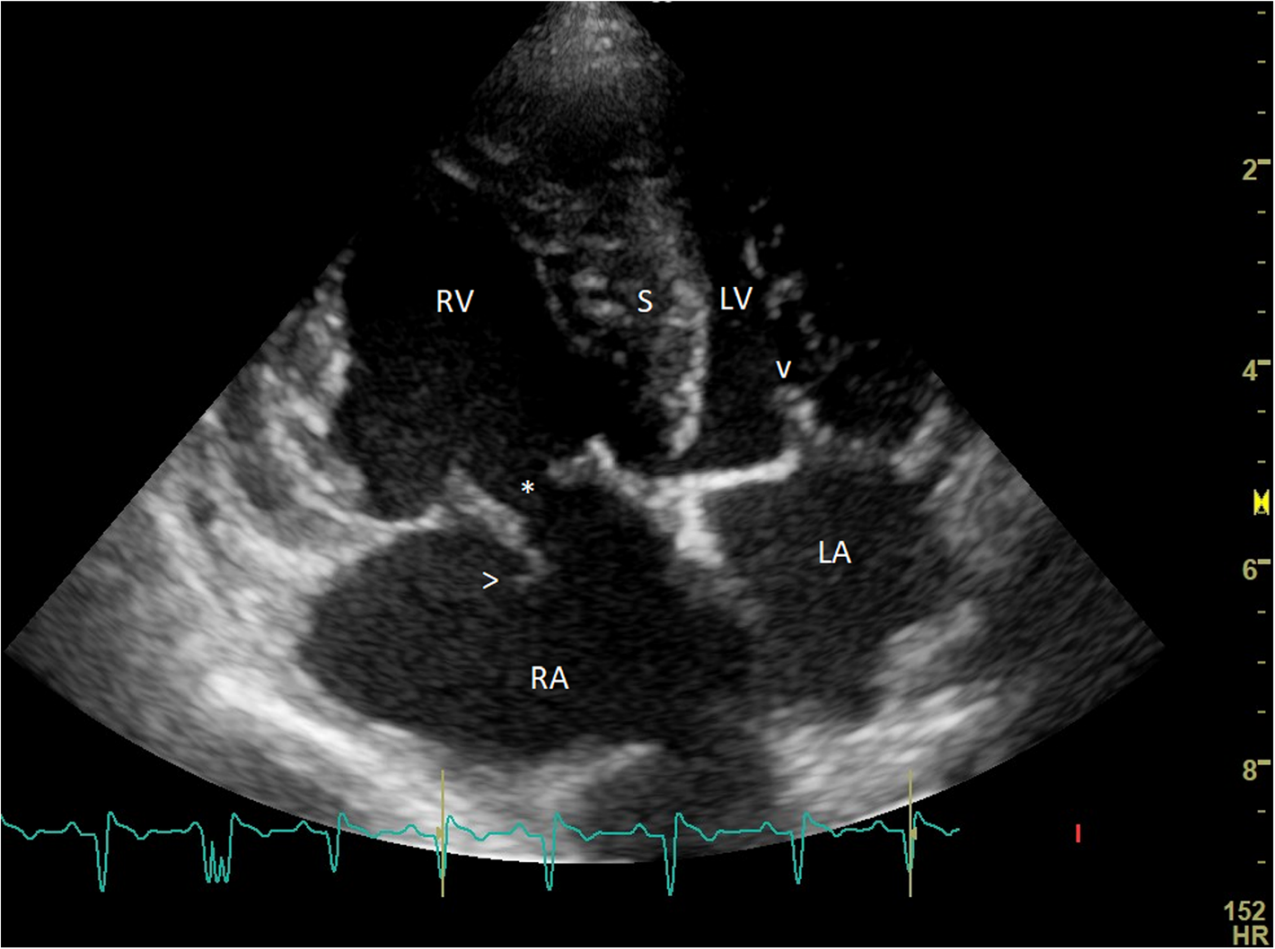
Two-dimensional echocardiographic image of the heart of a dog with severe pulmonary hypertension and ruptured chordae tendineae of the tricuspid valve. A 4-chamber view from the standard left parasternal caudal window. On the left of the image is the right side of the heart with the right atrium (RA) on the bottom and the right ventricle (RV) on the top of the image. On this systolic frame, the flail anterior leaflet of the tricuspid valve can be appreciated in the right atrial lumen causing lack of valvular coaptation, whereas the mitral valve is closed (v). Asterix (*) indicates the opening between the tricuspid valve leaflets. On the flail leaflet, remnants of ruptured chords can be recognized (>). In addition, severe right atrial dilatation, compared to the normal sized left atrium (LA) can be appreciated. The right ventricle shows a severe concentric and eccentric hypertrophy (thickened wall and dilated lumen), whereas the left ventricular lumen (LV) is small, and the wall shows pseudohypertrophy because of underfilling. S = interventricular septum. Synchronous ECG shows a sinus rhythm with negative QRS-complexes, presumably because of a right-sided deviation of the mean electrical axis as a result of right ventricular hypertrophy

Transthoracic echocardiography was performed using 2-dimensional, M-mode, color Doppler, pulsed wave Doppler and continuous wave Doppler techniques via the standard views [5, 12]. Echocardiographic examination confirmed the previously suspected severe pulmonary hypertension. A trivial amount of pericardial effusion was visible without echocardiographic signs of cardiac tamponade. The right ventricle showed severe concentric and eccentric hypertrophy. There was severe tricuspid valve regurgitation present with a systolic right ventricular to right atrial pressure gradient of 135 mmHg (reference < 31 mmHg) determined with continuous wave Doppler technique using the simplified modified Bernoulli equation [3, 5, 13, 14]. Two-dimensional echocardiography disclosed a flail anterior leaflet of the tricuspid valve. On this leaflet, chordal remnants could be recognized (Fig. 1). The pulmonary trunk and the left and right pulmonary arteries showed severe uniform dilation (pulmonary trunk to aortic ratio of 1.53; reference: 0.80-1.15) and a physiologic regurgitation jet [5, 15]. The pulmonary artery velocity profile evaluated with pulsed wave Doppler echocardiography showed rapid rise of flow with an acceleration time of 38 ms (ref. mean 93 ± 16 ms) [14]. The right atrium was moderately enlarged and the interatrial septum showed bulging towards the left (Fig. 1) [5]. Severe systolic and diastolic flattening and paradoxical movement of the interventricular septum were noticed, which led to a severely decreased left ventricular lumen size in diastole (normalized diastolic left ventricular internal diameter of 0.62; reference: 1.27-1.85) and in systole (0.26; reference: 0.71-1.26) (Fig. 1) [5, 16]. The left atrium was of normal size with a left atrium to aortic ratio of 1.4; ref. </=1.6) [5, 17, 18]. No mitral regurgitation was noticed. The mitral inflow pattern showed an E-wave of 0.74 m/s (reference 0.52-0.81 m/s) and a tall A-wave of 1.79 m/s (reference 0.45-0.78 m/s) on pulsed wave Doppler examination [5, 19, 20]. The calculated pressure half-time from the mitral E-wave velocity profile was 80 ms, which was prolonged (ref. < 50 ms), suggesting a mitral valve stenosis [19, 20]. The systolic flow velocities within the pulmonic trunk and the aorta were within the reference ranges (1.0 and 1.2 m/s, respectively) [5, 15]. Focused abdominal ultrasonography showed a large amount of ascites and a marked diffuse hepatomegaly with congested hepatic veins and distended caudal vena cava without any respiratory caliber changes [5]. Synchronous ECG showed sinus rhythm with positive P-waves and negative QRS-complexes.

Baermann larval isolation from a mixed fecal sample of three consecutive days was performed, which revealed a large number of L1 larvae of *Angiostrongylus vasorum* [1]. Fecal sedimentation and flotation techniques were negative for parasitic eggs. A serological test for circulating antigens (Angio Detect®, IDEXX) was performed from a blood sample, which was still positive. The daily oral furosemide and weekly milbemycin oxime therapies were stopped, and oral fenbendazole (50 mg/kg SID for 14 days) and oral sildenafil (1.4 mg/kg BID) tablets were prescribed and the dog was discharged. A re-check examination was scheduled in 6 weeks’ time.

At the 6 weeks re-check examination the owners reported resolution of all previously noted clinical signs, such as abdominal distension, cough and exercise intolerance. Physical examination revealed a bright, alert and responsive dog, with a body condition score of 6 out of 9. The dog lost 2.6 kg in this period and weighed 14.5 kg. Abdominal distension was no longer apparent. The respiratory rate was 40 breaths/min, and the breathing pattern was costo-abdominal. The femoral pulse was moderately powerful, regular, symmetric and without a pulse deficit with a frequency of 92/min. The mucous membranes were pink with a capillary refill time less than 1 second. A grade 2 out of 6 systolic cardiac murmur was auscultated over the right cardiac apex as well as a 1 out of 6 systolic murmur with the point of maximal intensity at the mitral valve region. Transthoracic echocardiography revealed no pericardial or pleural effusion. The right ventricle still showed a severe concentric and eccentric hypertrophy. A severe tricuspid valve regurgitation was still present with a systolic right ventricular to right atrial Doppler-derived pressure gradient of 90 mmHg. The previously recognized flail tricuspid valve leaflet was still present. The pulmonary trunk and the left and right pulmonary arteries still showed a severe uniform dilation without any abnormal content (such as heartworms or thrombus). The pulmonary trunk to aorta ratio decreased (1.19) compared to the initial examination. The pulmonary artery velocity profile was unchanged with an acceleration time of 40 ms (ref. mean 93 ± 16 ms) [3, 13, 14]. The pulmonic valve showed an unchanged physiologic regurgitation jet [5, 7]. The right atrium was still moderately enlarged and the interatrial septum bulged towards the left atrium. Flattening of the interventricular septum was present, but was less severe than at the initial examination. The left ventricular lumen size in diastole was now normal (normalized diastolic left ventricular internal diameter of 1.29; reference: 1.27-1.85) [16]. The left atrium was of normal size with a left atrium to aortic ratio of 1.5; ref. </=1.6) [17, 18]. The mitral valve showed a diastolic doming and a moderate systolic central regurgitation jet [5, 19]. The mitral inflow pattern consisted of an E-wave of 1.08 m/s and an A-wave of 2.47 m/s. In addition, the E-wave showed an even longer pressure half-time (100 ms, ref. < 50 ms) than at the initial examination [19, 20]. Focused abdominal ultrasound examination no longer showed any peritoneal effusion. The liver was still diffusely enlarged, and the gallbladder wall was diffusely thickened, consistent with edema. The hepatic veins and the caudal vena cava were subjectively markedly distended, similarly to the initial examination.

Baermann larval isolation test from a 3-day mixed fecal sample was performed, and revealed no larvae. A serological test for circulating antigens was repeated from a blood sample, and was negative. Continuation of the daily oral sildenafil (1.7 mg/kg BID) therapy was recommended until the next re-check in 2 months, and the dog was discharged. At the same time, monthly preventive moxidectin spot-on was advised for life-long use [21].

Two months later the owner reported via e-mail that the dog was doing excellent. Both the exercise tolerance and the abdominal size had normalized. The sildenafil was stopped 2 weeks prior to this e-mail contact, after gradual tapering of the daily dose. The owners decided against another re-check examination. However, a week later the owner reported recurrence of the abdominal distension via e-mail. An examination by the referring veterinarian confirmed the recurrence of ascites. Re-starting the sildenafil therapy (1.7 mg/kg, BID) resulted in resolution of this clinical sign within a week. After several months, the owner reduced the daily dosage of sildenafil (0.9 mg/kg, BID) due to financial reasons, which again led to recurrence of abdominal distension due to ascites. Afterwards, the daily dose was increased again (1.7 mg/kg, BID), which led to resolution of the abdominal distension. The dog was reported to be clinically healthy by the owners with daily oral sildenafil (1.7 mg/kg BID) administration 6 months after the date when eradication of French heartworm infection was confirmed with laboratory tests at the author’s institution. Thereafter, recurrence of ascites took place despite unchanged sildenafil therapy.

## Discussion and conclusions

The present case reports severe regurgitation and ruptured chordae tendineae of the tricuspid valve in a dog with severe pulmonary hypertension resulting from a French heartworm infection. The most common cause of a pathological primary tricuspid valve regurgitation in adult dogs is chronic myxomatous valve degeneration [22]. However, this condition leads to congestive heart failure much less frequently than degeneration of the mitral valve. Another possible cause of primary tricuspid valve regurgitation is congenital tricuspid valve dysplasia [5]. Secondary (or functional) tricuspid valve regurgitation in dogs can be caused by severe pulmonary hypertension, pulmonic stenosis and dilated or arrhythmogenic cardiomyopathies [4, 5, 23]. The underlying mechanism of tricuspid valve regurgitation in pulmonary hypertension in humans is thought to be related to the secondary eccentric right ventricular hypertrophy, which leads to tethering and tenting of the tricuspid valve leaflets, which, in turn, results in loss of valvular coaptation [24, 25]. Pure concentric hypertrophy of the right ventricle (as caused by severe congenital pulmonic stenosis), a condition that causes congenital pressure overload of the right ventricle, does not typically lead to tricuspid valve regurgitation [23].

Infection with *Angiostrongylus vasorum* is a well-documented cause of (precapillary) pulmonary hypertension in dogs [2, 3, 26–29]. The underlying mechanism of pulmonary hypertension is increased vascular resistance caused by the combination of vascular luminal obstruction and perivascular inflammation [3]. Pulmonary hypertension caused by this parasite can be partially or fully reversible, or irreversible [2, 3, 26, 29]. The underlying mechanism why the pulmonary hypertension in certain dogs persists despite eradication of the French heartworm infection remains to be determined. The persistence of pulmonary endarteritis, as it is reported in dogs after *Dirofilaria immitis* infection, or development of irreversible (such as plexiform) vascular lesions, might play a role, but histological studies in dogs with irreversible pulmonary hypertension after French heartworm infection are lacking [30–35].

In the present case report two conditions were found that might have potentially contributed to the pulmonary hypertension, a French heartworm infection and a congenital stenosis of the left atrioventricular valve [3, 5, 19]. Because the left atrium was of normal size, pulmonary hypertension of postcapillary origin (i.e. caused by the mitral stenosis) can be excluded [3, 20]. The “worsening” of the mitral stenosis at the re-check echocardiographic examination was the result of improved filling conditions of the left side of the heart, resulting from the decreased severity of the pulmonary hypertension [19, 20].

In humans, spontaneous rupture of the tricuspid valve apparatus is very rare, and it is thought to be associated with trauma or hypoxia of the papillary muscles secondary to pulmonary hypertension [10]. The latter mechanism might have played a role in the pathogenesis of chordal rupture in the present case. Severe hypoxemia was unlikely in this dog, however, because of the physiological respiratory rate and the lack of severe respiratory signs at presentation, when the clinical signs were the worst. Arterial blood gas analysis was not performed in this patient because of a risk for bleeding complications resulting from the (subclinical) coagulopathy that might accompany *Angiostrongylus vasorum* infection [1, 27]. As a consequence, in the present dog, a number of other conditions that could cause pulmonary hypertension were not actively excluded, such as pulmonary thromboembolism and diffuse interstitial pulmonary diseases (such as idiopathic pulmonary fibrosis). No laboratory diagnostic tests were performed to rule out dirofilariasis either, as the dog had not been to heartworm endemic areas. The presence of coinciding myxomatous tricuspid valve degeneration could not be ruled out in this dog, which might have contributed to the tricuspid valve regurgitation and the chordal rupture. However, the English Cocker Spaniel is not a breed predisposed to this condition, and if this disease was present, thicker leaflets and an involvement of the mitral valve would have be expected on the echocardiogram [5, 8, 9, 18, 22].

Though milbemycin and moxidectin are registered products for therapy of French heartworm infection in Europe the author instead chose daily oral fenbendazole therapy [1, 36]. Milbemycin is known to reduce the number of worms after 4 weeks of weekly oral therapy, but this was considered too long for this dog with very severe, life-threatening cardiac changes [1]. Moxidectin spot-on in dogs with severe French heartworm infection has anecdotally been associated with mortality [Glaus T, personal communication 2013 and the author’s unpublished observation]. For these reasons, daily fenbendazole therapy for 2 weeks was prescribed, and is known to be effective but does not lead to presumed anaphylactic reactions by the rapid death of the worms, as it is suspected with moxidectin [36].

Despite of the persistence of severe tricuspid regurgitation after successful eradication of the parasites, the ascites disappeared without any diuretic therapy or abdominocentesis. Because of the underfilled left ventricle and the suspected associated decreased cardiac output identified echocardiographically, sildenafil, a pulmonary arterial vasodilator, was the first choice as an additional therapy [3]. Sildenafil is a phosphodiestherase V inhibitor, and has vasodilatory effects specifically on the pulmonary artery branches. It has also been reported to have positive effects in dogs with severe pulmonary hypertension as a palliative therapy [3].

In conclusion, the rupture of major chordae tendineae of the tricuspid valve apparatus leading to a flail leaflet can be a complication of severe pulmonary hypertension in dogs. Despite of the persistence of severe tricuspid valve regurgitation and severe pulmonary hypertension after eradicating the French heartworms, the dog was kept free from clinical signs of congestive right-sided heart failure with long term daily administration of oral sildenafil.

## Data Availability

Not applicable.

## Abbreviation

ECG: electrocardiogram
